# Modulation by Melatonin of the Pathogenesis of Inflammatory Autoimmune Diseases

**DOI:** 10.3390/ijms140611742

**Published:** 2013-05-31

**Authors:** Gu-Jiun Lin, Shing-Hwa Huang, Shyi-Jou Chen, Chih-Hung Wang, Deh-Ming Chang, Huey-Kang Sytwu

**Affiliations:** 1 Department of Biology and Anatomy, National Defense Medical Center, No. 161, Section 6, MinChuan East Road, Neihu, Taipei City 114, Taiwan; E-Mail: lingujiun@mail.ndmctsgh.edu.tw; 2 Department of General Surgery, Tri-Service General Hospital, No.325, Section 2, Chenggong Rd., Neihu District, Taipei City 114, Taiwan; E-Mail: doc20333@yahoo.com.tw; 3 Graduate Institute of Medical Sciences, National Defense Medical Center, No. 161, Section 6, MinChuan East Road, Neihu, Taipei City 114, Taiwan; E-Mails: pedneuchen@hotmail.com (S.-J.C.); chw@ms3.hinet.net (C.-H.W.); 4 Department of Pediatrics, Tri-Service General Hospital, No.325, Section 2, Chenggong Rd., Neihu District, Taipei City 114, Taiwan; 5 Department of Otolaryngology—Head and Neck Surgery, Tri-Service General Hospital, No.325, Section 2, Chenggong Rd., Neihu District, Taipei City 114, Taiwan; 6 Institute of Undersea and Hyperbaric Medicine, National Defense Medical Center, No. 161, Section 6, MinChuan East Road, Neihu, Taipei City 114, Taiwan; 7 Department of Microbiology and Immunology, National Defense Medical Center, No. 161, Section 6, MinChuan East Road, Neihu, Taipei City 114, Taiwan; 8 Rheumatology/Immunology/Allergy, Tri-Service General Hospital, No.325, Section 2, Chenggong Rd., Neihu District, Taipei City 114, Taiwan; E-Mail: ming0503@ms3.hinet.net

**Keywords:** melatonin, autoimmune disease, multiple sclerosis, systemic lupus erythematosus, rheumatoid arthritis, type 1 diabetes mellitus, inflammatory bowel disease

## Abstract

Melatonin is the major secretory product of the pineal gland during the night and has multiple activities including the regulation of circadian and seasonal rhythms, and antioxidant and anti-inflammatory effects. It also possesses the ability to modulate immune responses by regulation of the T helper 1/2 balance and cytokine production. Autoimmune diseases, which result from the activation of immune cells by autoantigens released from normal tissues, affect around 5% of the population. Activation of autoantigen-specific immune cells leads to subsequent damage of target tissues by these activated cells. Melatonin therapy has been investigated in several animal models of autoimmune disease, where it has a beneficial effect in a number of models excepting rheumatoid arthritis, and has been evaluated in clinical autoimmune diseases including rheumatoid arthritis and ulcerative colitis. This review summarizes and highlights the role and the modulatory effects of melatonin in several inflammatory autoimmune diseases including multiple sclerosis, systemic lupus erythematosus, rheumatoid arthritis, type 1 diabetes mellitus, and inflammatory bowel disease.

## 1. Introduction of Melatonin

### 1.1. The Discovery, Biosynthesis and Biological Function of Melatonin

In 1917, McCord and Allen found that a substance extracted from bovine pineal gland could whiten the skin of *Rana pipiens* and named this substance melatonin (*N*-acetyl-5-methoxytryptamine). Melatonin is a product of the amino acid tryptophan. Tryptophan taken up by cells is first hydroxylated by tryptophan hydroxylase and then decarboxylated by decarboxylase, resulting in the formation of 5-hydroxytryptamine (serotonin). Serotonin is then *N*-acetylated by *N*-acetyltransferase (NAT) to form *N*-acetylserotonin. The final enzyme in the process of melatonin synthesis is hydroxyindole-*O*-methyltransferase (HIOMT), which *O*-methylates *N*-acetylserotonin and forms melatonin.

Melatonin is the major secretory hormone produced by the pineal gland during the night and has multifunctional properties [1]. It regulates circadian and seasonal rhythms [2,3], and is an effective antioxidant and scavenger of free radicals [4–6]. In the liver, melatonin is degraded enzymatically to 6-hydroxymelatonin [7]. As part of the process of scavenging free radicals and reactive species, this indoleamine generates other metabolites including cyclic 3-hydroxymelatonin, which is generated when melatonin scavenges two highly toxic hydroxyl radicals [8]. Melatonin is also secreted by neuroendocrine cells in the gastrointestinal tract (GIT), and its antioxidant action plays a role in protection of gastrointestinal mucosa from ulceration [9]. The effect of melatonin in the GIT has been suggested to include protection of the pancreas from acute pancreatitis [10]. Furthermore, an earlier study found that NAT and HIOMT, two key enzymes in the synthesis process of melatonin, are expressed in various extrapineal tissues of Sprague-Dawley (SD) rats, including kidney, muscle, testis and the organs of central nervous systems. NAT mRNA is also presented in the spleen while HIOMT is undetectable in this organ [11]. Gomez-Corvera *et al.* also demonstrated that mRNA of NAT is expressed in thymus, spleen, bone marrow and peripheral blood mononuclear cells (PBMCs) of mice, and the presence of melatonin is detectable in these tissues [12]. Carrillo-Vico *et al.* found that human PBMCs express NAT and HIOMT, and these enzymes are capable of synthesis of melatonin [13]. Moreover, there are studies that have reported the synthesis of melatonin in the thymus and bone marrow of humans [14,15]. The presence of, either enzymes involved in the synthesis of melatonin, or melatonin itself, in the immune tissues suggests a role of melatonin in the immune system.

### 1.2. The Effect of Melatonin on the Immune System

Recent studies have confirmed that melatonin plays an important role in the immune system [16]. Melatonin receptors are expressed on the membrane of CD4 T cells, CD8 T cells, and B cells [17,18]. It has been reported that the proliferation of T cells increases in mice treated with melatonin [19]. Melatonin treatment has also been reported to enhance the production of natural killer (NK) cells and monocytes in the bone marrow of mice [20], and can induce cytokine production in human peripheral blood mononuclear cells via the nuclear melatonin receptor [21]. By contrast, other studies have demonstrated that the expression of interleukin (IL)-2 and interferon (IFN)-γ is decreased and the expression of T helper (Th)2 cell cytokines, such as IL-4 and IL-10, is upregulated in mice treated with melatonin [22–24].

An antiproliferative effect of melatonin on lymphocyte-derived tumor cells has also been described. Raghavendra *et al.* demonstrated that melatonin inhibits the proliferation of 3DO.54.8-Th1-hybridoma cells by downregulating IL-2 secretion in these cells [24]. Majewska *et al.* suggested that melatonin suppresses cell-mediated immune responses partly through inhibiting the production of IL-12 in antigen-presenting cells (APC) [25]. Konakchieva *et al.* also reported that melatonin inhibits Concanavalin A-induced [^3^H]-thymidine incorporation in human peripheral blood lymphocytes and tonsillar lymphocytes [26]. Thus, melatonin can have dichotomous effects in the immune system by either activating or suppressing immune cells.

### 1.3. The Effect of Melatonin on the Production of Proinflammatory Cytokines

The effect of melatonin on the suppression of proinflammatory cytokine production has been proved in several earlier studies. Raghavendra *et al.* [24] demonstrated that melatonin suppresses the production of tumor necrosis factor (TNF)-α. Wang *et al.* [27] also demonstrated that melatonin decreases the production of proinflammatory cytokines including TNF-α and IL-1β from Kupffer cells in fibrotic rats. Melatonin also protects against experimental reflux esophagitis by repressing the upregulation of TNF-α, IL-1β, and IL-6 [28]. Nitric oxide has been found to be an important mediator in inflammatory response [29]. Melatonin also plays a role in the regulation of nitric oxide synthesis [30]. Previous study has shown that melatonin inhibits the expression of inducible nitric oxide synthase (iNOS) in liver and lung of lipopolysaccharide (LPS)-treated rat [31]. The *in vivo* study presented by Jung *et al.* also observed that melatonin intraperitoneal (i.p.) administration (50 mg/kg) in rats suppresses the mRNA expression of TNF-α, IL-1β, IL-6 and iNOS [32]. Veneroso *et al.* also found that melatonin administration at a lower dose (1 mg/kg, i.p.) decreases the mRNA levels of proinflammatory cytokines and protein level of iNOS and cyclooxygenase-2 (COX-2) in rats induced with cardiac inflammatory injury by acute exercise [33]. Furthermore, the protective role of melatonin in mitochondria dysfunction has been documented. Melatonin treatment prevents mitochondrial impairment and inhibits inducible mitochondrial NOS activity in septic mice [34,35]. The study presented by Lowes *et al.* also supports that melatonin reduces the production of IL-6 and IL-8, and prevents the loss of mitochondrial membrane potential in endothelial cells treated with LPS plus peptidoglycan G (PepG) [36]. Recently, they further proved that *in vivo* melatonin i.v. administration results in the reduction of serum IL-6 and the improvement of mitochondrial function in LPS plus PepG-induced acute sepsis in rats [37]. The mechanism of melatonin in the reduction of proinflammatory cytokine as well as iNOS production has been suggested via the inhibition of either expression or activation of nuclear factor-κB (NF-κB).

### 1.4. The Suppressive Effect of Melatonin on the Activation of NF-κB

NF-κB activation initiates the expression of genes involved in the inflammatory responses, such as proinflammatory cytokines, iNOS, adhesion molecules, COX-2, and matrix metalloproteinases (MMPs) [38]. Earlier studies have demonstrated that melatonin reduces the transcriptional activity and DNA binding of NF-κB by preventing its translocation to the nucleus [39]. This results in the reduction of proinflammatory cytokine and chemokine production. The expression of adhesion molecules regulated by NF-κB is also suppressed by melatonin [40], resulting in reduced recruitment of neutrophils to inflamed sites [41,42]. Gilad *et al.* demonstrated that melatonin suppresses the expression of iNOS in murine macrophages via suppression of NF-κB [43].

Recently, many studies confirmed that melatonin exerts anti-inflammatory effect through inhibition of NF-κB. Some of these studies found that the expression level of NF-κB was suppressed by melatonin treatment [44–47], while others suggested that melatonin mainly disturbed the translocation of NF-κB. In the *in vitro* studies, Qin *et al.* demonstrated that melatonin (10 μM) inhibits IL-1μ-induced expression and activation of MMP 9 in human umbilical vein endothelial cells (HUVECs) by suppressing NF-κB expression [48]. By contrast, Xia *et al.* revealed that melatonin treatment at 1 mM concentration attenuates LPS-induced upregulation of cyclooxygenase-2 (COX-2) and iNOS expression and inhibits the translocation of NF-κB in RAW264.7 cells via modulation of toll-like receptor 4-mediated signaling pathways [49]. The research in human chondrocyte cell line (CHON-001) also supports that melatonin (10 ng/mL) blocks hydrogen peroxide-induced production of iNOS by suppressing the degradation of IκB-α and the translocation of NF-κB [50]. However, Choi *et al.* demonstrated that the effect of melatonin in NF-κB is not that of reducing the degradation of IκB-α, but blocking the DNA binding activity of NF-κB p50 subunit in LPS-activated RAW264.7 cells [51]. A study has found that melatonin inhibits p300 histone acetyltransferase (HAT) activity and the acetylation in NF-κB p50 subunit, which may result in the reduction in DNA binding activity of NF-κB [52]. Shi *et al.* further demonstrated that melatonin treatment markedly inhibits the binding of NF-κB to COX-2 and iNOS promoter. This effect probably results from the reduction of p300 HAT induced-acetylation in NF-κB p50 [53].

In the *in vivo* studies, the effect of melatonin in the suppression of NF-κB action via inhibition of its translocation or binding activity has been identified [32,33,54]. The study presented by Kang *et al.* demonstrated that melatonin i.p. administration (10 mg/kg) attenuates the protein and mRNA expression levels of TNF-α, IL-6 and iNOS in the liver of SD rat with ischemia and reperfusion injury. They also observed that the translocation of NF-κB was reduced and the cytosolic level of IκB-α was decreased [55]. Furthermore, the treatment dosage of melatonin may also critical to the anti-inflammatory effect. The study presented by Wang *et al.* demonstrated that melatonin treatment at pharmacological concentration (1 mM), but not at physiological concentration (1 nM), inhibits the activation of NF-κB in T98G and U251 glioma cells [56].

Generally, melatonin administration at pharmacological level suppresses the production of inflammatory cytokines and iNOS via inhibition of NF-κB. The underlying mechanisms may go through inhibition of NF-κB expression, reduction of IκB-α degradation and NF-κB translocation, or suppression of the DNA binding activity of NF-κB.

### 1.5. The Modulatory Effect of Melatonin on the Th1/2 Balance

Melatonin has been reported to exert immunomodulatory effects on Th1/2 development. The study reported by Garcia-Maurino *et al.* found that melatonin enhances the production of IL-2, IL-6, and IFN-γ in human circulating CD4 cells [17]. It has been observed that the peak plasma levels of melatonin in healthy subjects coincide with a peak in the secretion of lipopolysaccharide-stimulated IFN-γ in whole blood [57]. These data imply an association between IFN-γ production and melatonin secretion. Melatonin can also activate Th1 lymphocytes by increasing IL-12 production by APC [58].

By contrast, melatonin plays a role in the enhancement of Th2 immunity. Shaji *et al.* have reported that treatment of BALB/c mice with melatonin induces T cells to secrete IL-4 and downregulates the levels of IL-2 and IFN-γ [23]. Some of these differences may be related to the different doses of melatonin used. Previous studies have demonstrated that treatment of mice with low-dose melatonin restores attenuated T cell activity [59] and increases Th1 cytokine levels [60]. By contrast, high-dose (200 mg/kg) melatonin treatment significantly decreased the immunoglobulin (Ig)M-response to allogeneic stimulation and abrogated acute rejection of cardiac allografts in rats, while low-dose (20 mg/kg) melatonin increased the production of allospecific IgM antibodies [61]. These observations suggest that melatonin may differentially modulate the immune responses in a dose-dependent manner *in vitro* and *in vivo*.

## 2. Overview of Autoimmune Diseases and Modulatory Effects of Melatonin on These Diseases

Autoimmune diseases affect approximately 5% of the population in Western countries. These diseases can be systemic, such as systemic lupus erythematosus (SLE), or organ-specific, such as type 1 diabetes mellitus (T1D). Autoimmune diseases are caused by the activation of immune cells, such as T or B cells, by antigens in the normal tissues. Activation of autoantigen-specific immune cells leads to subsequent damage to target tissues by these activated cells [62].

In the next section of this article, we will discuss the role and the modulatory effect of melatonin in several inflammatory autoimmune diseases including multiple sclerosis (MS), SLE, rheumatoid arthritis (RA), T1D, and inflammatory bowel disease (IBD).

### 2.1. Multiple Sclerosis

#### 2.1.1. The Pathogenesis and Animal Model of MS

MS is the most prevalent inflammatory demyelinating disease of the central nervous system (CNS) in human adults [63]. The prevalence varies from 60–200 per 100,000 in Northern Europe and North America to 6–20 per 100,000 in lower-risk areas. This disease results from the loss of the neuronal myelin sheath because of attack by autoantigen-specific immune cells, and usually manifests at between 20 and 40 years of age [64]. Experimental autoimmune encephalomyelitis (EAE) is the most frequently used animal model of MS because of its clinical and histopathological similarities to MS [65]. It can be induced in several species of animals by immunization with whole myelin proteins or specific myelin peptide epitopes, including myelin oligodendrocyte glycoprotein or myelin basic protein, emulsified in complete Freund’s adjuvant [66].

Both adaptive and innate immune cells are associated with the pathogenesis of MS. T cells and macrophages were observed to infiltrate the CNS lesions in animals with EAE [67]. The adaptive immune CD4 T cells play a critical role in the initiation of the autoimmune process. Innate immune cells such as macrophages produce inflammatory cytokines under stimulation by CD4 T cells. The presence of proinflammatory cytokines in CNS contributes to the severe inflammation seen in EAE and results in damage to the myelin sheath and neurons. These cytokines also lead to the generation of reactive oxygen species in the affected sites. Indeed, markers of oxidative stress have been reported to be increased in the sera of MS patients [68–70] and in the CNS of rats with hyperacute EAE [71].

#### 2.1.2. The Role of Melatonin in MS

An association between melatonin and MS has been suggested by several clinical observations. Sleep disruption is a frequent complaint in MS patients and contributes to daytime fatigue [72]. Melamud *et al.* observed that MS patients excrete a subnormal proportion of 6-sulpahtoxymelatonin (a melatonin metabolite) in the urine at night, indicating a dysregulation of melatonin production or altered enzymatic metabolism of melatonin in MS patients, and may suggest melatonin may be involved in this disease [73]. Shift work leads to disrupted circadian rhythms and sleep restriction, and a report published in 2011 found that shift work at a young age is associated with an increased risk for MS [74]. These reports suggest that a dysregulation of physiological level of melatonin may contribute to the pathogenesis of MS.

#### 2.1.3. The Modulatory Role of Melatonin in EAE

The application of melatonin for the treatment of MS has been examined in the EAE animal model of this disease. One study in EAE indicated that melatonin treatment (5 mg/kg for 15 days) inhibited the onset of disease and reduced the severity of clinical signs. This protective effect resulted from the suppression of intracellular adhesion molecule (ICAM)-1 production in the spinal cord [75]. However, an earlier study demonstrated that inhibition of the immunoenhancing effects of melatonin by luzindole, a melatonin receptor antagonist, prevented the onset of EAE [76]. Luzindole acts as a selective melatonin receptor antagonist, with a higher affinity for the MT2 receptor than for the MT1 receptor [77]. Since both MT1 and MT2 are expressed on the cell surface of lymphocytes, this selective antagonist may trigger a complex effect in lymphocytes that could underlie the contrasting conclusions of these two animal studies.

### 2.2. Systemic Lupus Erythematosus

#### 2.2.1. The Pathogenesis and Animal Model of SLE

SLE is an autoimmune disease resulting from damage to tissues by immune cells. This disease may manifest at any age and in either sex, but women are more frequently affected than men. SLE is characterized by overproduction of a variety of anti-nuclear autoantibodies. Common symptoms include malar (“butterfly”) rash, photosensitivity, nephritis, and arthritis [78].

The pathogenesis of SLE involves activation of autoreactive T cells that subsequently initiate the hyperactivity of B cells. The activation of B cells leads to polyclonal hypergammaglobulinemia and immune complex deposition. Either Th1 or Th2 immune responses can be elevated in this disease. The levels of Th1 cytokines such as IL-2 and IFN-γ, and of Th2 cytokines, mainly IL-4, are increased in patients affected by SLE [79]. However, SLE is still considered a Th1-dominant disease [80]. In addition, the inflammatory cytokines TNF-α and IL-6 also contribute to the disease, and the importance of the IL-23-Th17 axis has also been demonstrated [81].

#### 2.2.2. The Dual Role of Melatonin in SLE Is Gender-Dependent in Animal Models

Observation of the serum melatonin level in MRL-*Fas^lpr^* mice found that the circadian melatonin rhythm was uncoupled from the light/dark cycle [82]. In the MRL-*Fas^lpr^* mouse model of SLE, IL-10 deficiency exacerbates the development of disease, including a reduction in the survival rate, increased severity of glomerulonephritis and skin lesions, enhanced production of pathogenic IgG2a/2b antibodies and an increased population of IFN-γ-producing T cells [83]. The results of this study strongly suggest a protective role of IL-10 in SLE. Thus, the effect of melatonin on the induction of IL-10 production may be beneficial for the prevention or therapy of SLE. In fact, Jimenez-Caliani *et al.* found that melatonin administration (30 mg/kg in drinking water for 1 month) reduced serum levels of autoantibodies, decreased the production of inflammatory cytokines, and increased the secretion of IL-10 in female MRL-*Fas^lpr^* mice. By contrast, in male mice, melatonin treatment shifted the Th2 immune response to a Th1 pattern that displayed higher levels of inflammatory cytokines and autoantibodies [84]. Their data indicated that administration of melatonin has a gender-dependent dichotomous effect in MRL-*Fas^lpr^* mice in the early stages of disease. A later study presented by the same authors confirmed that this gender-dependent effect of melatonin in MRL-*Fas^lpr^* mice is through modulation of sex hormones [85].

The application of melatonin treatment also has a beneficial effect in another animal model of SLE. Treatment of mice with pristane-induced lupus with various doses of melatonin (0.01, 0.1, 1.0 mg/kg) reduced the serum titers of anti-single-stranded DNA and anti-histone antibodies. Histopathological analysis showed that this treatment also reduced the renal injury, resulting in milder glomerular atrophy and less thickening of capillary walls. The secretion of IL-6 in splenic lymphocytes was also reduced in melatonin treated mice [86]. The authors suggested that this beneficial effect in the pristane-induced lupus mouse model is through the regulation of cytokine disturbances. Wu *et al.* also showed that melatonin treatment (20 mg/kg, sc.) ameliorates murine membranous nephritis through its antioxidant, antiapoptotic, and immunomodulatory effects [87]. The results of this study imply that melatonin treatment may also act to ameliorate nephritis in SLE. Based on these studies, melatonin treatment could be beneficial for therapy in SLE.

### 2.3. Rheumatoid Arthritis

#### 2.3.1. Pathogenesis and Animal Model of RA

RA is a chronic inflammation of the joints characterized by progressive erosion of cartilage and bone that is associated with the formation of proliferated pannus. The most commonly used animal model of this disease is collagen-induced arthritis (CIA) in which immune cells, such as CD4 T cells and macrophages, infiltrate the joints of experimental animals and lead to the inflammation and damage of joints.

The pathogenesis of RA is associated with hyperplasia, increased vascularity, and infiltration of inflammatory immune cells to the synovial membrane of patients. Autoantigen-specific CD4 T cells are a major contributor to the initiation of this autoimmune response. Activated autoreactive CD4 T cells stimulate monocytes, macrophages, and synovial fibroblasts to produce inflammatory cytokines, such as IL-1β, IL-6, and TNF-α, which drive inflammation in RA. CD4 T cells also stimulate B cells to generate immunoglobulins related to this disease, such as rheumatoid factor [88].

#### 2.3.2. The Disease-Promoting Effect of Melatonin in RA

RA patients suffer from joint pain and morning stiffness. It has been suggested that this is associated with the influence of the hormonal effect of circadian rhythms. In RA patients, serum melatonin levels exhibit a wider plateau than in healthy people [89]. The serum levels of proinflammatory cytokines, especially IL-6, exhibit a close association with stiffness and pain levels in patients with RA, in whom the peak serum levels of TNF-α and IL-6 occur at 6:00 am and 7:00 am, respectively [90]. By contrast, these two cytokines reach peak values at 3:00 am and 6:00 am, respectively, in healthy people.

Androgens have been found to exert a protective effect against the development of RA [91]. In the study presented by Valenti and Giusti, melatonin receptor was presented in the plasma membrane of isolated rat Leydig cells, and the secretion of testosterone in these cells was reduced in the presence of melatonin [92]. This effect has been suggested to be the cause of the lower incidence of RA in men. A clinical trial of melatonin treatment in RA has been undertaken [93], in which RA patients received 10 mg melatonin at night in addition to ongoing medication. There was an increase in the erythrocyte sedimentation rate and neopterin concentrations in melatonin-treated patients, suggesting a proinflammatory effect of melatonin. However, there were no significant effects on clinical symptoms or on the concentrations of IL-1β, IL-6, and TNF-α in the serum of melatonin-treated patients.

#### 2.3.3. Investigation of Melatonin in an Animal Model of RA

The influence of melatonin on the onset of RA has also been investigated in an animal model of this disease. An earlier study reported that constant darkness exaggerates the development of CIA in DBA/1 mice, implying a pathogenic role of melatonin in CIA [94]. Hansson *et al.* demonstrated that DBA/1 mice injected subcutaneously with 1 mg/kg melatonin once daily showed exaggerated development of CIA via enhancement of T cell priming [95]. In addition, pinealectomy ameliorates CIA in either DBA/1 or NFR/N mice [96]. As melatonin is the major product of the pineal gland, this result suggests that unlike the other autoimmune diseases we discuss in this article, melatonin may have a promoting effect in the development of RA. Although one study found that melatonin possesses dose-dependent (from 1 to 100 μg/kg) prophylactic and therapeutic effects in an adjuvant-induced arthritis rat model [97], a recent study documented that melatonin treatment (10 mg/kg) increased the severity of CIA, probably via attenuation of the expression of *cryptochrome 1* [98]. These contrasting observations may result from the different dosages or different models (DBA/1 mouse *vs.* rat) used in these studies. A study published in 2005 that used a CIA rat model and a lower dosage of melatonin treatment (30 μg/kg) demonstrated that melatonin had a proinflammatory effect and increased the severity of joint damage [99]. Thus, these previous studies in animal models of RA suggest that melatonin trends to promote the development or increase the severity of RA.

### 2.4. Type 1 Diabetes Mellitus

#### 2.4.1. Pathogenesis of T1D

T1D, also known as insulin-dependent diabetes mellitus (IDDM), results from the destruction of insulin-producing β cells in the pancreatic islets and has been identified as a T cell-mediated autoimmune disease [100]. T1D is usually diagnosed in young people and is also called as juvenile-onset diabetes or childhood-onset diabetes. People with T1D have the classical phenotype (polydipsia, polyphagia, and polyuria) of diabetes mellitus resulting from hyperglycemia-induced osmotic dieresis and secondary thirst. This disease is characterized by hyperglycemia (resulting from abnormally increased gluconeogenesis and insufficient glucose disposal), ketosis (resulting from the accumulation of free fatty acids and their oxidation), insulitis (mononuclear cell infiltration of islets), and the presence of anti-islet antibodies (including anti-insulin antibodies (AIAs), IA-2 autoantibodies, and anti-glutamic acid decarboxylase (GAD) antibodies).

#### 2.4.2. Pathogenesis of Autoimmune Diabetes in NOD Mice

The NOD mouse strain spontaneously develops T cell-dependent β cell destruction resembling human T1D, and serves as an animal model for this autoimmune disease [101]. This mouse strain exhibits a sexual dimorphism not seen in humans, where 80%–90% of female mice, but only 40%–50% of male mice, develop diabetes by the age of 30 weeks [102]. Treatment of female NOD mice with androgen at 8 weeks of age prevented islet destruction and diabetes without eliminating the islet inflammatory cells [103]. These observations indicate an association between the endocrine and immune systems.

It has been well characterized that an imbalance between Th1 and Th2 responses predisposes NOD mice to developing autoimmune diabetes [104,105]. The CD4 T cells in NOD mice show a Th1-cell-dominant phenotype, which manifests as increased IFN-γ and decreased IL-4 production by activated CD4 T cells of NOD mice [106]. It has been identified that Th1 cells play a pathogenic role in initiation of the disease process: treatment of NOD mice with neutralizing antibodies against IFN-γ can effectively prevent the onset of diabetes [107], and adoptive transfer of IFN-γ-depleted cells can prevent diabetes in NOD mice [108]. In addition, administration of typical Th2 cytokines, IL-4 or IL-10, can prevent or delay the onset of autoimmune diabetes [109,110].

#### 2.4.3. The Influence of Melatonin on the Insulin Production of Pancreatic β Cells

The expression of melatonin receptors has been detected in pancreatic β cells [111]. Thus, melatonin treatment may also influence the physiological activities of β cells such as insulin production. Indeed, a study has demonstrated that the synthesis of melatonin was increased in an animal model of streptozotocin-induced T1D [112]. Moreover, an influence of melatonin on insulin secretion has also been documented. Stumpf *et al.* reported that melatonin treatment inhibits insulin secretion in rat INS1 insulinoma β cells [113]. However, Ramracheya *et al.* demonstrated that melatonin may have a species-specific effect [114]; melatonin treatment inhibited glucose-stimulated insulin secretion by mouse insulinoma β cells (MIN6), but stimulated insulin secretion in human islets. Another possibility to explain these differences may be the differences between insulinoma β-cell lines and primary isolated islets. The insulinoma β cell lines are composed of a single cell population and primary isolated islets contain not only β cells, but also α-cells, δ-cells, and Schwann cells. The different composition of β cell lines and islets may explain the diverse results of these studies.

#### 2.4.4. Modulatory Role of Melatonin in T1D

T1D has been considered a Th1-cell-dominant autoimmune disease. Thus, the effect of melatonin in shifting the immune response toward Th2 cells may be beneficial for disease modulation. Furthermore, it is well documented that proinflammatory cytokines such as TNF-α and IFN-γ contribute to β cell destruction. The immune cells infiltrating in the islets, such as macrophages and CD8 T cells, secrete the proinflammatory cytokines TNF-α and IL-1β, which enhance inflammation and induce apoptosis of β cells [100]. Therefore, the anti-inflammatory effect of melatonin may also be beneficial for the inhibition of disease onset or improvement of the survival of islet grafts transplanted for T1D therapy. Indeed, a study has shown that melatonin treatment prolongs the lifespan of NOD mice whereas neonatal pinealectomy accelerates the development of autoimmune diabetes [115].

#### 2.4.5. The Application of Melatonin on Islet Transplantation

Islet transplantation has been established as a potential therapeutic strategy for T1D that could provide nearly perfect blood glucose monitoring and modulation. Recurrent autoimmunity plays a crucial role in islet graft destruction in NOD mice [116,117] and in human islet transplantation [118,119]. Moreover, destruction of grafts by autoimmune recurrence often takes place earlier than allogeneic graft rejection [120]. Suppression of autoimmune recurrence and allograft rejection is therefore a critical issue in islet transplantation.

The effects of melatonin in organ transplantation have also been explored. Several studies indicated that melatonin treatment prolongs graft survival in solid organ transplantation. Sapmaz *et al.* [121] demonstrated that a single i.p. melatonin injection after autologous ovary transplantation attenuated ovarian tissue necrosis following engraftment. In addition, high-dose (200 mg/kg) melatonin treatment prolonged the survival of cardiac allografts in rats by reducing lymphocyte proliferation [61]. Our previous work showed that high-dose melatonin treatment effectively prolonged the survival of islet grafts in diabetic NOD recipients [122], and showed increased expression of the immunosuppressive cytokine IL-10 and an increased population of IL-10-producing CD4 T cells in the spleen of melatonin-treated NOD mice. These results may in part explain the reduced proliferative ability of T cells and the decreased population of Th1 cells in mice treated with high-dose melatonin, and are consistent with a previous study by Raghavendra *et al.* showing that melatonin treatment increases the amount of IL-10 in serum [22]. In the same report, this group also demonstrated that melatonin treatment increased the production of IL-10 by anti-CD3-stimulated T cells in the presence of APC [24]. It has also been reported that IL-10 treatment prolongs the survival of grafts in islet transplantation. Moreover, Zhang *et al.* [123] demonstrated that adeno-associated virus-mediated IL-10 gene transfer inhibits autoimmune recurrence in syngeneic islet cell transplantation in NOD mice. Kim *et al.* [124] also reported that transduction of IL-10 into islets combined with subtherapeutic doses of cyclosporine significantly prolonged islet allograft survival. Our study confirmed that melatonin treatment elicits a protective effect in islet transplantation of NOD mice via IL-10. These results support the concept that melatonin treatment may be of benefit in either syngeneic or allogeneic islet transplantation for the treatment of T1D.

### 2.5. Inflammatory Bowel Disease

#### 2.5.1. Pathogenesis of IBD

Crohn’s disease and ulcerative colitis are immunologically mediated disorders collectively referred to as IBD. They are characterized by idiopathic, chronic, and relapsing inflammation in the small and large intestines. However, these two types of colitis also exhibit several different features. For example, the inflammation in Crohn’s disease is usually transmural, whereas in ulcerative colitis it is typically confined to the mucosa. Crohn’s disease usually affects the ileum and colon, but also other regions of the intestine, in a discontinuous pattern. By contrast, ulcerative colitis affects the rectum and part of or the entire colon with an uninterrupted pattern [125]. The most frequently used animal models for this disease are chemically induced colitis in rats or genetically modified in mice. The chemically induced models include trinitrobenzene sulfonic acid (TNBS)-induced colitis, dextran sulfate sodium (DSS) colitis and oxazolone colitis. The genetically modified models include IL-10-deficiency colitis, T-bet transgenic mice, and STAT4 transgenic mice [126].

Accumulation of immune cells in the intestinal tissues of patients is one characteristic of IBD. The cells infiltrating the lamina propria include innate and adaptive immune cells, such as neutrophils, macrophages, dendritic cells, B cells, and T cells. Activation of these immune cells in the intestinal mucosa induces inflammatory responses and elevates the local levels of cytokines including TNF-α, IL-1β, IL-6, and IFN-γ [127]. Effector T cells play a critical role in the pathogenesis of IBD. Th1 and Th2 cells are involved in the development of Crohn’s disease and ulcerative colitis, respectively [128]. T cells isolated from the lamina propria lesions of Crohn’s disease produce increased amounts of IFN-γ, indicating a Th1 phenotype. By contrast, T cells from ulcerative colitis produce increased amounts of IL-5, suggesting an atypical Th2 inflammation [129]. In addition, recent genetic association analysis studies of IBD patients or investigations in murine models of this disease have demonstrated that the IL-23-Th17 pathway also plays an important role in the pathogenesis of IBD [130–133].

#### 2.5.2. The Therapeutic Potential of Melatonin Administration in IBD

The effect of melatonin treatment in animal models of IBD has been investigated. A study in 1995 showed that daily i.p. melatonin administration (150 μg/kg) reduced the severity of DSS colitis in mice [134]. It was also reported that pretreatment of rats with melatonin (5 mg/kg and 10 mg/kg) reduced the disease scores for colonic inflammation induced by acetic acid [135]. This result is consistent with a previous study documenting that melatonin treatment at the same dose significantly ameliorated colonic injury in rat models of acetic acid-induced colitis and TNBS-induced colitis [136]. Cuzzocrea *et al.* also showed that melatonin treatment (15 mg/kg daily, i.p.) significantly reduced the appearance of diarrhea and the loss of body weight in rats with dinitrobenzene sulfonic acid-induced colitis [137]. This therapeutic effect is mediated by decreasing the production of TNF-α, reducing the activation of NF-κB, decreasing the expression and activity of matrix metalloproteinase (MMP)-9 and -2, and the antiapoptotic effect of melatonin [138,139]. The effect of melatonin administration has also been tested in a TNBS-induced colitis rat model. Treatment with10 mg/kg/day i.p. melatonin injection for 15 days significantly decreased the pathological disease scores in TNBS-treated rats and reduced the expression of NF-κB and the activities of myeloperoxidase, malondialdehyde, and caspase-3 in colon tissue. By contrast, the colonic level of glutathione was increased by this treatment. The results of this study suggest that melatonin administration is beneficial for IBD via its antioxidant, anti-apoptotic, and antiinflammatory effects [140–142]. This result has also confirmed by another study in the same year, where acute administration of 2 mg/kg melatonin significantly reduced the severity of colitis in TNBS-treated rats although long-term treatment had a negative influence on disease outcome [143].

#### 2.5.3. Clinical Trial of Melatonin Treatment in IBD

The application of melatonin in IBD patients has also been documented. Chojnacki *et al.* have evaluated adjuvant treatment with melatonin in ulcerative colitis patients. This strategy kept the treated patients in remission for the 12 months of their observation period. The C-reactive protein concentration in the blood of melatonin-treated patients remained within the normal range during the course of the study, and the decrease in hemoglobin concentration in melatonin-treated patients was less than that in placebo controls [144]. These results indicate that adjuvant melatonin therapy should be helpful in sustaining remission in ulcerative colitis patients.

## 3. The Future Application of Melatonin Treatment in Autoimmune Diseases

Melatonin has been demonstrated to possess multiple activities, including antioxidation, anti-inflammatory, and immunomodulatory effects. Therefore, this natural pineal gland product is highly attractive to investigators of autoimmune diseases. For example, IL-10 is one of the most extensively investigated and promising candidates for the treatment of autoimmune diseases [145,146], and IL-10-deficient NOD mice show accelerated development of diabetes under cyclophosphamide treatment [147]. However, treatment of NOD mice with recombinant human IL-10 prevents the onset of T1D [110]. IL-10-deficient mice spontaneously develop chronic enterocolitis [148], which can be prevented by transferring IL-10-producing CD4 T cells [149]. Given the ability of melatonin to elicit IL-10 production and increase the number of IL-10-producing CD4 T cells [150–152], melatonin treatment might be applicable for the therapy of autoimmune diseases such as MS, SLE, and IBD.

One major concern in these studies we discussed in this review is the different routes of melatonin administration. In oral administration, melatonin is first metabolized by liver. By contrast, intraperitoneal or subcutaneous injection may bypass hepatic metabolism in the beginning of melatonin treatment. An earlier study presented by Kennaway and Seamark showed that subcutaneous injection of melatonin quickly leads to a high plasma level in sheep, but it declines rapidly. By contrast, oral administration leads to a lower but constant plasma melatonin level [153]. DeMuro *et al.* demonstrated that intravenous injection of melatonin can reach a much higher serum melatonin concentration in humans when compared to oral administration. However, the half-life of melatonin in the serum showed no significant difference between these two different routes of administration [154]. These results suggest that the routes of melatonin administration will influence the peak plasma level of melatonin but not the half-life of melatonin. Therefore, administration of melatonin by intravenous or subcutaneous injection will be suitable in the clinical application of melatonin treatment for higher dosages.

Another concern in the application of melatonin treatment for autoimmune diseases is the high doses used in the experiments with animal models. However, melatonin exhibits a high level of safety in clinical trials. In a clinical trial in patients with amyotrophic lateral sclerosis, a daily oral dose of 30–60 mg of melatonin was well tolerated and generated no side effects [155]. In a later clinical trial, patients who received daily rectal melatonin treatment at a higher dose (300 mg per day) also did not show any complications [156]. A recent report also demonstrated that treatment of patients undergoing major aortic surgery with up to 60 mg intravenous melatonin during surgery was safe and without complications [151]. Therefore, application of a high pharmacological dose of melatonin in the therapy of autoimmune diseases should be acceptable. However, the treatment doses used in some of the animal studies are higher than the treatment of melatonin used in human clinical trials. Thus, the appropriate doses used in clinical application for inflammatory autoimmune diseases still need to be examined.

## 4. Concluding Remarks

**Figure 1:**
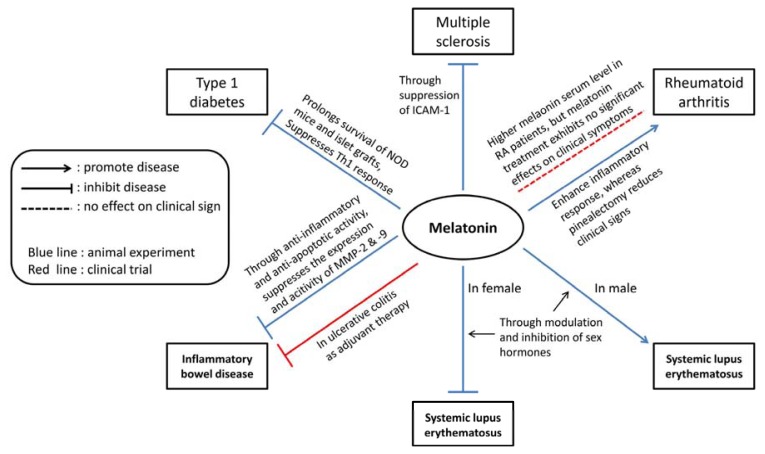
The possible modulatory roles of melatonin in animal models and clinical trials of inflammatory autoimmune diseases.

Melatonin therapy has been studied in several animal models of autoimmune disease and evaluated in patients with clinical autoimmune disease (Figure 1). For most of the autoimmune diseases discussed in this article except RA, melatonin treatment reduced the severity of disease in animal models (Table 1) and some clinical trials [93,144]. These observations suggest the importance of endogenous melatonin in the development of autoimmune disease and the possibility of exogenous melatonin treatment of human autoimmune diseases.

## Figures and Tables

**Table 1:** The effects of melatonin in the animal model of autoimmune diseases.

Clinical diseases	Animal models	Melatonin treatment	Results	Reference
Multiple sclerosis	EAE	Receptor antagonist (luzindole)	Suppresses the onset of EAE	[76]
	EAE	5 mg/kg, orally	Reduces the incidence and severity of clinical signs	[75]
Systemic lupus erythematosus	MRL/MpJ-*lpr^Fas^* mice	30 mg/kg, dw	Ameliorates disease in females, whereas exacerbates disease in males	[84]
	MRL/MpJ-*lpr^Fas^* mice	30 mg/kg, dw, combined with testosterone in females, with estradiol in males	Decrease in total serum IgG, IgM, anti-ddsDNA, and anti-CII autoantibodies titers	[85]
	pristane-induced lupus in Balb/c mice	0.01, 0.1, 1.0 mg/kg, intragastric	Display a beneficial effect on disease	[86]
	Membranous nephritis in Balb/c mice	20 mg/kg, sc.	Suppresses the pathological injury of glomeruli and deposition of immune complexes	[87]
Rheumatoid arthritis	CIA in DBA/1 mice	Constant darkness	Exaggerates severity and chronicity of arthritis	[94]
	CIA in DBA/1 mice	1 mg/kg, sc.	Increase arthritis severity	[95]
	CIA in DBA/1 and NFR/N mice	Pinealectomy	Ameliorates arthritis in both strain	[96]
	AA in SD rats	1, 10, 100 μg/kg, intragastric	Ameliorates arthritis and inhibits inflammatory response	[97]
	CIA in Wistar rats	30 μg/mouse, sc.	No significant effect in hitopathologic features, increases the levels of IL-1β and IL-6 in serum	[99]
	CIA in DBA/1 mice	10 mg/kg, i.p.	Increases paw thickness and joint destruction	[98]
Type 1 diabetes mellitus	NOD mice	pinealectomy	Promotes disease onset	[115]
	NOD mice	4 mg/kg, sc.	Protects from the development of disease	[115]
	NOD mice	200 mg/kg, sc.	Prolongs islet grafts survival in NOD recipient	[122]
Inflammatory bowel disease	DSS-induced colitis in mice	150 μg/kg, i.p.	Reduces the severity of colitis	[134]
	DNBS-colitis in SD rats	15 mg/kg, i.p.	Reduces severity of colitis	[137]
	TNBS and acetic acid-colitis in SD rats	5 and 10 mg/kg	Protects colonic injury in both colitis models	[136]
	TNBS-colitis in SD rats	2.5, 5, 10 mg/kg	Reduces colonic inflammatory injury	[141]
	TNBS-colitis in SD rats	2.5, 5, 10 mg/kg, intracolonic	Attenuates colonic injury	[142]
	TNBS-colitis in Wistar-albino rats	10 mg/kg, i.p.	Decreases colitis scores	[140]
	DNBS-colitis in SD rats	15 mg/kg, i.p.	Attenuates colonic injury	[138]
	TNBS-colitis in Wistar rats	acute administration: 0.5, 1, 2 mg/kg, i.p.; chronic administration: 1 and 2 mg/kg, i.p.	Short-term administration protects from colitis while chronic administration aggravates colitis	[143]
	Acetic acid-colitis in Wistar rats	10 mg/kg, i.p. and intracolonic	Protects from colitis induced colonic damage	[135]
	DNBS-colitis in SD rats	15 mg/kg, i.p.	Reduces colonic injury	[139]

## Abbreviations

EAE: experimental autoimmune encephalomyelitis
CIA: collagen-induced arthritis
AA: adjuvant-induced arthritis
i.p.: intraperitoneal injection
sc.: subcutaneous injection
dw: drinking water
SD: Sprague-Dawley
NOD: non-obese diabetic
DSS: dextran sodium sulphate
DNBS: dinitrobenzene sulfonic acid
TNBS: trinitrobenzene sulfonic acid
